# Nanostructured Biomaterials for Tissue Repair and Anti-Infection

**DOI:** 10.3390/nano12091573

**Published:** 2022-05-06

**Authors:** Jiajun Qiu, Xuanyong Liu

**Affiliations:** State Key Laboratory of High Performance Ceramics and Superfine Microstructure, Shanghai Institute of Ceramics, Chinese Academy of Sciences, Shanghai 200050, China; qiujiajun@mail.sic.ac.cn

Biomaterials play a vital role in regenerative medicine, aiming to regenerate and replace lost/dysfunctional tissues. For instance, biomaterials are widely used for joint replacement, dental implants, orthopedic fixations, stents, and so on. Once biomaterials are implanted into the human body, there are inevitable interactions between biomaterial surfaces and the biological environment, such as proteins, cells, and bacteria. Therefore, implant failures still happen due to the implant-related complications resulting from poor implant integration, infections, mechanical instability, inflammation, etc. [1]. Thus, more efforts need to be put into addressing these challenges. 

Many nanostructures can be found in nature. For example, the surface of lotus leaves is composed of fine-branched nanostructures that show super-hydrophobicity [2]. Aligned nanocolumns with diameters of about 70 nm and a column-to-column distance of about 90 nm exist in cicada wings, which endows cicadas with a self-cleaning property [3]. Additionally, nanostructures can be found in the human body. For example, human bones are composed of nanosized organic and mineral phases [4]. This indicates that nanostructures show promise in the field of biomaterials. Nanotechnology is a powerful tool in modern materials science and is able to incorporate biomimicry on the nanoscale into biomaterials and endow them with bioactivity [5]. 

Bacterial infection is one of the major post-surgery complications in clinics. Antibiotics have been considered as effective weapons against bacterial infections. However, antibiotic resistance is presently an emerging public health threat, caused by the overuse and abuse of antibiotics. Therefore, it is urgent to develop new methods to combat bacteria. In the past decade, mechanical and physical sterilization has attracted considerable attention, which does not cause bacterial resistance, as the morphology of nanometers can directly cause bacterial death through physical and mechanical interactions [6].

Based on this, various types of nanostructured biomaterials have been developed and many surface modification techniques have been adopted to produce nano-functionalized biomaterials surface. Due to the advantages of nanoscale features, nanostructured biomaterials show enhanced biocompatibilities, such as cell adhesion, proliferation, and differentiation, and present excellent antibacterial activity. These nanostructured biomaterials exhibit promising applications in biomedical fields.

This Special Issue, “Nanostructured Biomaterials for Tissue Repair and Anti-infection”, focuses on the latest research progress of nanostructured biomaterials for tissue repair and anti-infection. Both original research articles and review articles covering the current progress on nanostructured biomaterials for tissue repair and anti-infection are welcomed. This Special Issue includes, but is not limited to, the following research topics, surface nano-functionalization of biomedical metals, ceramics, polymers for tissue repair, and anti-infection.
